# Does Allulose Appeal to Consumers? Results from a Discrete Choice Experiment in Germany

**DOI:** 10.3390/nu14163350

**Published:** 2022-08-16

**Authors:** Kristin Jürkenbeck, Theresa Haarhoff, Achim Spiller, Maureen Schulze

**Affiliations:** 1 Marketing for Food and Agricultural Products, Department for Agricultural Economics and Rural Development, University of Goettingen, Platz der Göttinger Sieben 5, 37073 Göttingen, Germany; 2 Department of Management, Society and Communication, Copenhagen Business School, Dalgas Have 15, 2000 Copenhagen, Denmark

**Keywords:** d-allulose, psicose, choice experiment, novel food, consumer preference, rare sugar

## Abstract

Reducing the sugar content in food is an important goal in many countries in order to counteract obesity and unhealthy eating. Currently, many consumers eat a number of foods with too much sugar content. However, mankind has an innate preference for sweet foods, and thus one strategy is to have food products which taste sweet but consist of a reduced calorie and sugar content. Allulose is a rare monosaccharide and is considered a safe ingredient in foods, for example in the US, Japan, Singapore, and Mexico, while in Europe, it is in the approval process as a novel food. Thus, it is relevant to find out how consumers perceive the different attributes of allulose in comparison to other sweeteners. Therefore, an online survey consisting of a choice experiment was conducted in Germany to find out consumer preferences of sweeteners. The survey data were analyzed using a mixed logit model. The results reveal that taste is the most important attribute for sweeteners, which explains about 40% of the choice. In the attribute level, a typical sugar taste is preferred. As allulose has a typical sugar taste, the likelihood that it appeals to consumers is high. The second most important attribute is the base product.

## 1. Introduction

Over the last few decades, the prevalence and incidence of diseases related to excessive weight gain, such as obesity, type 2 diabetes, and hypertension, have increased worldwide. For example, globally, the number of people who are living with diabetes has nearly doubled between 1980 (4.7%) and 2014 (8.5%) [1]. Those incidences have been linked to excessive sucrose intake. Dietary choices are a key factor that influence this global burden [2]. The World Health Organization (WHO) published a guideline that suggests limiting the intake of sugar to 10% of the daily total energy intake [3,4]. They even recommend to further reduce the sugar intake to 5% [4]. Reducing the sugar content in foods is an important goal in many countries, e.g., Belgium, Italy, UK, and Germany [5]. To achieve this goal, there are many national projects to promote healthy eating. Examples are the ‘healthy weight strategy’ in the UK [6] or the ‘national reduction and innovation strategy’ in Germany [7].

The preference of mankind for sweetness is innate before birth [8]. Exposure to sweet beverages and sugary foods during childhood leads to an increased preference for sweet tastes [9]. In addition, nutrition is a habitual behavior and difficult to change [10]. Therefore, one strategy is to use sweeteners with similar characteristics (e.g., taste and processing properties) as the common sugar called sucrose, but with fewer calories. Depending on the level of sweetness in relation to sucrose (international reference of sweetness potency = 1), sweeteners are classified into two groups, namely nutritive and non-nutritive sweeteners. Until now, most nutritive sweeteners have had a similar or lower sweetening potency as sucrose, but these sweeteners do not lower the calorie content of food substantially [11]. One exception is, for example, erythritol, which is a non-caloric sweetener [12]. Non-nutritive sweeteners have a much higher sweetening potency. Thus, only small quantities are necessary to achieve the required sweetening level, and therefore the calorie content added to the food is low [13].

Within the category of sweeteners, stevia is a natural non-nutritive sweetener, while xylitol and erythritol are low-caloric sugar alcohols [14]. Xylithol and erythritol are sourced from natural origin. Stevia, xylitol, and erythritol are already available in supermarkets and contained in foods, while allulose is not available in Germany. Stevia has, compared to sugar, about 300 times the sweetening potency and a liquorice-like taste of its own [15]. The consumption of stevia causes no known consequences for human health as long as it lies within the limit of 4 mg/kg of body weight/day [16]. There is no known effect on blood glucose levels, and stevia does not cause caries and is calorie-free [17]. Xylitol, also known as birch sugar, is not usually made from birch wood but from agricultural residues such as corncob pomace, straw, and hardwoods. It is not caries-causing and has about half the calories of conventional sugar. The sugar alcohol xylitol does not affect insulin levels, but in larger quantities it can have a laxative effect [18]. Erythritol occurs naturally only in small quantities in fruits, vegetables, and cheese and therefore mostly comes from industrial production [19]. In various biotechnological processing steps with the help of fungi, erythritol is produced from carbohydrates such as glucose or sucrose. Erythritol does not promote caries, is calorie-free, and does not affect insulin levels, but in larger quantities it can have a laxative effect [18].

Allulose (also known as d-allulose, psicose) is a novel rare sugar. Allulose occurs naturally, for example in figs, kiwis, and raisins, but only in very small quantities. Using enzymes, the rare monosaccharide allulose can be obtained from conventional beet sugar or maize through enzymatic conversion [20]. As a sugar, allulose has similar functional properties as conventional sugar. It is available in crystalline powder and is easily dissolved in water [21]. The sweetness of allulose is about 70% that of sucrose. Due to its low calorific value of 0.4 kcal/g [22], it has just 10% of the calories of sucrose. Allulose melts at 90 °C and forms caramel [23]. Additionally, allulose has many health advantages in comparison to conventional sugar, including no influence on blood glucose levels (glycemic control). Furthermore, diets supplemented with allulose are suggested to prevent obesity and diabetes [23]. One main advantage of allulose is that it has a typical sugar taste [23], and sometimes a hint of caramel is associated with it. In 2012, the US Food and Drug Administration approved allulose (GRN No. 400) as safe. Thus, it is allowed to be used as an ingredient in a variety of foods and nutrition supplements in the US. Allulose has also received approval in Mexico, Japan, Singapore, and South Korea [24]. In Europe, allulose is currently in the approval process at the European Food Safety Authority (EFSA), as it falls under the novel food regulation as a previously unavailable food.

To learn more about the market potential of sweeteners, it is important to investigate consumer preferences of sweeteners. German consumers perceive erythritol as artificial (88%) and stevia as in-between artificial (49%) and natural (51%). Only xylitol is perceived as natural, by 65% of consumers (the researcher in this study also mentioned the German name ‘Birkenzucker’, or ‘birch sugar’ in English, which may have led to a naturalness bias) [25]. Polish consumers said that the main reason to limit the sugar intake was due to weight control. To limit the amount of sugar in their diet, they used sweeteners instead [26]. In Germany, one study found out that ‘fewer calories’ is seen as the most important benefit of sweeteners [25]. A review about natural sweeteners revealed that consumers are eager to eat foods consisting of natural ingredients but do not want to compromise on taste [15]. In line with that is the research on Canadian consumers who judge taste and naturalness as the most important point of consideration when buying sugars or sugar substitutes [27]. Moreover, frozen desserts containing natural non-nutritive sweeteners did not fulfill the expectations of consumers. Consumers judged taste as more important than perceived healthiness [28]. Another study showed that consumers’ healthiness perception of sweeteners varied widely. High fructose corn syrup (64%) and aspartame (52%) are perceived as less healthy than table sugar, while almost half of the consumers perceived raw sugar (48%) as healthier than table sugar [29]. In their research, Goodman et al. [29] concluded that the perceived level of naturalness and not the calorie content might be related to consumers’ perception of sweetener healthiness. Moreover, consumers indicated that they wanted to reduce their intake of sugar compared to low calorie sweeteners [29]. Additionally, sweeteners are perceived as risky [30]; thus, parents think sweeteners are not safe for their children to consume [31], which affects the acceptance of sweeteners [30].

In the marketing of sweeteners, a wide variety of characteristics could be used in communication. Conceivable topics include, for example, calories, naturalness, taste, or health aspects. So far, it is not known which of these characteristics consumers prefer or which characteristics consumers look for when buying sweeteners. These insights are of particular importance in the context of reformulating food recipes and therefore help food producers to decide which sweetener to use in their food products. Sweeteners are important in terms of reformulation because they can reduce the calorie content and make food products healthier, for example, more tooth-friendly. As an approval by the EFSA of allulose is expected in Europe, it is necessary to analyze which properties of sweeteners are important for consumers. To analyze consumer preferences regarding different characteristics of four sweeteners (allulose, stevia, xylitol, and erythritol), an online survey including a choice experiment was conducted. The data were analyzed using a mixed logit model. The results will allow companies to tailor their communication strategy based on consumer preferences. This is especially important for companies who want to sell or use allulose in their foods. Allulose is considered as an important ingredient in food in the future. The global market size is projected by 2027 to reach US $27 million [32].

## 2. Materials and Methods

### 2.1. Methodology of the Discrete-Choice Experiment and Experimental Design

In discrete-choice modeling, it is assumed that individuals choose from a set of products in order to maximize their utility [33]. The decision maker must select one alternative among a given choice set [34]. Part-worth utilities for each product attribute can be determined to identify important determinants [35]. Choice experiments have been validated for various products to simulate consumers’ purchase behavior [36,37,38,39,40,41].

**Figure 1:**
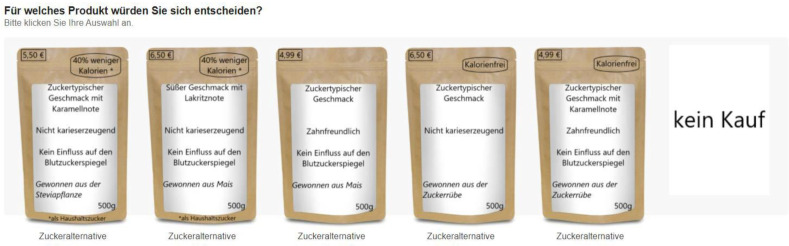
Example of a choice set.

An online survey including a hypothetical choice experiment was conducted in November 2020 in Germany. In total, 440 respondents received the questionnaire through an online panel provider (Respondi AG, Cologne, Germany). Only respondents who indicated that they follow a healthy or very healthy diet could take part in the survey. Participation in the survey was voluntary. Participants provided their electronic consent in the beginning of the online survey. They could withdraw from participation by closing the browser or survey. All participants were informed that data would be anonymously analyzed and that no data can be identified and/or linked to individual participants. Further, quotas for gender, age, and income were used to reflect the German population. Some respondents (38) were excluded from the data set due to excessively rapid response behavior (below ½ of the average response time). Thus, 403 respondents remained in the data set for analysis. In the beginning, respondents had to answer questions about their own diet and their food shopping behavior, which was followed by the choice experiment. Before choosing between the product alternatives, consumers were shown an explanation of the included sweeteners to ensure consistent knowledge among all participants (Appendix A). IBM SPSS 26 was used to create an orthogonal reduced factorial design resulting in 25 product alternatives. Thus, five choice sets of five product alternatives and a no-purchase option were included. The no-purchase option enables consumers to not choose any of the alternatives if they do not fulfill their preferences. An example of a choice set can be seen in Figure 1.

The product alternatives in each choice set were randomized in order and sequence, while the no-purchase option was always presented last. The level selection was based on the characteristics of four sweeteners: allulose, stevia, erythritol, and xylitol. The choice of the selected sweeteners was grounded on strategic competitive decisions of a sweetener manufacturer. The price levels were based on market prices (Rewe, Edeka, and Kaufland) in Germany at the time of questionnaire preparation; €4.99 and €6.50 per 500 g packs were the lowest and highest prices on the market. The respective attributes and levels are shown in Table 1.

To analyze the data of the choice experiment, Stata 16.0 (College Station, TX, USA) was used, while descriptive statistics were performed using IBM SPSS 26.0 (Armonk, NY, USA). Effect coding was used for all attributes. The no-information level was the base effect-coded level. In addition, the no-purchase option was modeled as the base dummy-coded level.

### 2.2. Ranking of the Allulose Attribute Levels

After completing the choice experiment, respondents had to order the attribute levels of allulose according to their preferences from most important to least important. In a preference ranking task, participants are forced to discriminate between the mentioned characteristics while at the same time revealing the degree of appreciation [42,43]. Ranking tasks have been compared to results from choice experiments previously [43,44,45]. The initial order of the allulose levels was presented in a randomized order for each participant. The ranking task was completed by participants by using a drag-and-drop function for the five allulose attribute levels. The number of participants who assigned a specific allulose level to a rank were multiplied by the corresponding place, meaning the level that ranked in the first place was multiplied by five, the second place by four, the third place by three, the fourth place by two, and the fifth place by one. Following this procedure, rank coefficients were calculated.

## 3. Results

### 3.1. Descriptive Statistics

The results showed that the sociodemographic variables of the sample reflect the German population in the characteristics gender, age, and education quite well (Table 2).

When purchasing food, respondents pay most attention to naturalness (3.96), regional production (3.86), low sugar (3.84), and few additives (3.81) (Appendix B, Table A1).

### 3.2. Results of the Choice Experiment

The results of the calculation of the attribute importance show that taste is by far the most important one. It is followed by the base product and the influence on the blood glucose level. Price, dental health, and calorie content were equally important (Table 3).

All four attribute levels of taste had a highly significant influence on consumer choice. The part-worth utilities for ‘typical sugar taste’ and ‘sweet taste’ are positive, while the part-worth utilities for ‘typical sugar taste with a hint of caramel’ and ‘sweet taste with liquorice note’ are negative, meaning that they are not attractive for consumers. The level of ‘typical sugar taste’ showed the overall highest part-worth utility (β = 0.85). The ‘base product’ of the extracted sugar alternative influenced consumers’ choice positively when it was ‘extracted from stevia plant’, while it was negative for ‘extraction from wood’. ‘Tooth-friendly’ as a level of dental health is attractive to consumers. Additionally, consumers value the attribute level ‘calorie-free’ of the attribute ‘calorie content’. None of the levels of ‘influence on blood glucose level’ nor ‘price’ had an influence on consumers’ decisions. For all attribute levels, the base alternative was the ‘no purchase’ option, meaning that a negative part-worth utility signals that consumers would rather choose not to purchase instead of an attribute level consisting of a negative part-worth utility (Table 4).

### 3.3. Results of the Ranking Task of Allulose Attribute Levels

The results of the ranking task show that the attribute level of allulose of ‘no influence on blood glucose level’ was most important to respondents, followed by ‘calorie-free’ and ‘not caries-causing’ (Table 5). This shows that consumers who can only consider the attribute levels of allulose evaluate them differently than when other sweetener alternatives are available.

## 4. Discussion

This research is the first work to examine the importance of allulose characteristics in comparison to three other sweeteners. The results reveal that taste is the most important attribute when purchasing sweeteners. This result is in line with earlier findings by Mintel [27]. A study which included frozen dessert (consisting of non-nutritive sweeteners) also showed that taste is more important than perceived healthiness [28]. Research of other food products also showed that taste has a major influence on choice [46,47]. The second-most important attribute is the base product. It is the source the sweetener is made of and suggests a hint of naturalness of the product. This result is in line with earlier research which reported that naturalness is an important aspect of foods [48]. It is interesting that the ‘influence on blood glucose level’ is the third-most important attribute. Price, dental health, and calorie content followed as being equally important to consumers. In comparison, earlier research showed that fewer calories are an important characteristic for consumers when choosing sweeteners [25]. This difference could be due to different data collection approaches. The results of the current research are based on hypothetical choice decisions, and thus relative importances, while the results from Ears and Eyes [25] are based on judgments on a five-point Likert scale.

The results indicate that a ‘typical sugar taste’ is the most preferred taste, followed by ‘sweet taste’. Research showed that a preference for sweet taste can be observed by all people independent of their age [49]. The other attribute levels, which consist of an aftertaste (caramel or liquorice), are not favored by consumers. The communication of attributes that further describes the sugar taste profile (e.g., hint of caramel) will not lead to a positive rating; even the wording ‘typical sugar taste with a hint of caramel’ has a negative rating. It is the attribute level with the overall highest negative part-worth utility. This clearly shows that consumers prefer a typical sugar taste or a sweet taste.

It is surprising that the ‘extracted from sugar beets’ level is not significant, as it is the vegetable from which sugar is often made. Research has shown that naturalness is an important aspect when buying foods [48]. Extracting ingredients from sugar beets for sugar production could fulfil the consumers’ desire for naturalness. Goodman et al. [29] concluded that naturalness is the aspect that consumers use when judging the healthiness of sweeteners and not the calorie content. In the current study, the stevia plant is the most-preferred base product. One possibility could be that the base product ‘stevia’ is also the product name with which it is sold and is therefore well-known by consumers. However, stevia has a sweet taste with a liquorice note, which in fact was not preferred by consumers. These contradicting results show that consumers might have very little knowledge about the production of sweeteners. Providing consumers with information about the production process could increase their acceptance of sweeteners from natural base products. Another explanation could be that consumers rate the base product ‘stevia plant’ as natural but do not prefer the taste of stevia.

‘Influence on blood glucose level’ is not significant. About 8 million Germans have diabetes, and a further 2 million have diabetes but are not diagnosed. It is expected that by 2040, around 12 million Germans will suffer from diabetes [50]. This currently makes up 10% (2040: 15%) of the German population who must pay attention to the influence on blood glucose level (glycemic control) when eating. Thus, it can be predicted that this level might gain importance in the near future. Allulose could be a good choice for glycemic control, as research showed that in healthy individuals, it does not raise blood glucose and insulin levels [51]. Further, if small quantities of allulose are added to high glycemic-index carbohydrate meals, it can lower the postprandial blood glucose in participants with prediabetes [52].

Price has no significant influence on consumers’ purchasing behavior of sweeteners. Other studies showed that price is one of the most important characteristics of food purchase [36,37,47,53]. It is possible that consumers assume that they only need small quantities of sweeteners, so that the price per use is low.

‘Tooth-friendly’ is the only level of dental health which is attractive for consumers. Research depicted that sugar consumption increases the risk for caries [54]. Therefore, society associates sugar consumption with caries. Phrases such as ‘no sweets for children, otherwise there will be caries’ could be anchored in people’s minds. Sweeteners such as allulose are tooth-friendlier [55]—a promising product characteristic to differentiate sweeteners from conventional sugar.

As the participants of this study only consisted of people who stated that they follow a healthy diet, trying to follow a healthy diet could be connected to dental health as well. Given that participants were following a healthy diet, the influence of the attribute ‘tooth-friendly’ could be lower in the German population at large.

In terms of the ‘calorie content’, only the level ‘calorie-free’ influences consumer decision-making. This is positive, as allulose, stevia, and erythritol were the sweeteners included that were nearly calorie-free. Since allulose has significantly fewer calories than regular sugar, this sweetener would help consumers in their calorie control [22]. This therefore demonstrates the potential for allulose to support consumers in their weight management. In earlier research, fewer calories were identified as the most important attribute of sugar alternatives [25]. Moreover, research showed that using calorie labeling promotes healthier food choices [56]. In this sense, allulose may help consumers to follow a diet consisting of a reduced number of calories.

When consumers ranked the attribute levels of allulose, the results were different. In the ranking task, ‘no influence on blood glucose level’ achieved the highest rank. This is different from the choice experiment where the level was not significant. Furthermore, the two levels of the base product and taste, which were the most important levels in the choice experiment, were the least important in the ranking task. These differences can be based on the fact that in the ranking task, the attribute levels of allulose were queried, whereas the attribute importance contains the attributes themselves. For example, the taste of allulose was also the least-preferred level within the attribute of taste in the choice experiment. Also, the price was not included in the ranking task.

This study is the first approach to analyze consumers preferences of allulose. One limitation is that the research consists of a hypothetical purchase decision, thus future studies should involve real purchases in a supermarket and consider monetary consequences. Second, only a limited number of sweeteners are included in the survey. Future studies should include cheap talk scripts to reduce hypothetical bias.

## 5. Conclusions

From a public health point of view, sugar consumption is too high in society. A society-wide change in consumption habits towards less-sweet and reduced-sugar-content foods is desirable to achieve the target of the WHO. As a preference for sweetness is innate in mankind, it is difficult to change it. Allulose has the potential to be a sweetener with characteristics that are important for German consumers. Taste is the most important attribute, followed by the base products. The taste of allulose is typical to sugar and achieved the highest importance. In terms of the base product, allulose is produced from a natural source and thus fulfills consumer desire for naturalness. In comparison to other sweeteners, allulose has several advantages, e.g., it has no influence on blood glucose levels and is tooth-friendly. Further, due to its low calorific value, it can help consumers with calorie control and weight management. Allulose could be applied in a number of product categories, for example in soft drinks, ketchup, sweets, or yogurts. As the results show that taste is the most important attribute, sensory studies should be conducted to analyze how consumers judge the allulose taste in a tasting.

## Figures and Tables

**Table 1:** Attributes and levels used in the choice experiment.

Attribute	Level
Taste	Typical sugar taste; Typical sugar taste with a hint of caramel; Sweet taste; Sweet taste with liquorice note; No information
Calorie content	Calorie-free; 10% fewer calories than table sugar; 40% fewer calories than table sugar; No information
Dental health	Tooth-friendly; Not caries-causing; No information
Influence on blood glucose level	No influence on blood glucose level; Low influence on blood glucose level; No information
Base product	Extracted from sugar beets; Extracted from stevia plant; Extracted from maize; Extracted from wood; No information
Price (€)	4.99; 5.50; 5.99; 6.50; No information

**Table 2:** Sample description.

Sociodemographic Variables	Sample (%)	German Population * (%)
Male	48.09	49.32
Female	51.91	50.68
15–25	8.38	8.86
26–40	18.90	22.05
41–65	45.45	44.09
65+	27.27	25.00
No graduation (yet)	1.44	4.10
Certificate of secondary education	27.75	30.90
General certificate of secondary education	33.49	30.90
General qualification for university entrance	16.03	15.00
University degree	21.29	19.19

Source: * Statistisches Bundesamt (2019): Statistisches Jahrbuch 2019, Wiesbaden.

**Table 3:** Attribute importance.

Attribute	Relative Importance (%)
Taste	40.03
Base product	18.48
Influence on blood glucose level	13.32
Price	9.72
Dental health	9.36
Calorie content	9.10

Note: Relative importances add up to 100%.

**Table 4:** Part-worth utilities of all levels.

Attributes and Levels	Part-Worth Utilities (β)	Std. Errs.	z
Taste			
Typical sugar taste with a hint of caramel	−0.87 ***	0.53	−1.65
Typical sugar taste	0.85 *	0.19	4.43
Sweet taste with a liquorice note	−0.75 *	0.26	−2.86
Sweet taste	0.50 *	0.13	3.74
Base product			
Extracted from sugar beets	0.49	0.39	1.25
Extracted from stevia plant	0.10 ***	0.06	1.62
Extracted from maize	−0.24	0.28	−0.85
Extracted from wood	−0.31 *	0.08	−3.67
Influence on blood glucose level			
No influence on blood glucose level	0.15	0.28	0.55
Low influence on blood glucose level	−0.42	0.50	−0.84
Price			
€4.99	0.01	0.12	0.06
€5.50	−0.08	0.10	−0.78
€5.99	0.18	0.10	0.67
€6.50	−0.24	0.15	−1.59
Dental health			
Tooth-friendly	0.18 **	0.08	2.14
Not caries-causing	−0.22	0.15	−1.55
Calorie content			
Calorie-free	0.18 *	0.09	2.03
10% fewer calories than table sugar	−0.21	0.14	−1.58
40% fewer calories than table sugar	0.13	0.20	0.67

Note: Log-likelihood: −3341.7086, base alternative: no-purchase option, Wald chi2 = 333.45, Prob > chi2 = 0.0000, *** *p* = 0.10, ** *p* = 0.05, * *p* = 0.01.

**Table 5:** Results of the ranking task of the allulose attribute levels.

Attributes	Attribute Levels	Ranking Values
Influence on blood glucose level	No influence on blood glucose level	720
Calorie content	Calorie-free	455
Dental health	Not caries-causing	360
Base product	Extracted from sugar beets	265
Taste	Typical sugar taste with a hint of caramel	185

Note: The number of participants who assigned a specific allulose level to a rank were multiplied by the corresponding place, meaning the level that ranked in first place was multiplied by five, the second place by four, the third place by three, the fourth place by two, and the fifth place by one. Following this procedure, rank coefficients were calculated.

## Appendix A

### Appendix A.1. German Version of the Information of the Four Sweeteners

Die Allulose ist ein kalorienfreier Zucker aus der Natur. Gewonnen wird die Allulose durch einen Veredelungsprozess des herkömmlichen Rübenzuckers aus regional angebauten Zuckerrüben. Dies geschieht mithilfe von Enzymen. Dennoch handelt es sich um ein reinpflanzliches Produkt. Die Allulose ist nachweislich zahnfreundlich und außerdem geeignet für Diabetiker, da sie den Blutzuckerspiegel sowie den Insulinspiegel nicht beeinflusst. Im Gegensatz zu vielen anderen Zuckeralternativen besitzt sie einen zuckertypischen Geschmack und ist gut verträglich.

Erythrit kommt natürlicherweise nur in geringen Mengen in Obst, Gemüse und Käse vor und stammt daher meist aus industrieller Produktion. In verschiedenen biotechnologischen Verarbeitungsschritten mit Hilfe von Pilzen wird Erythrit aus Kohlenhydraten wie Glukose oder Saccharose hergestellt. Die Zuckeralternative ist nicht Karies fördernd und kalorienfrei. Erythrit belastet nicht den Insulinspiegel, kann in größeren Mengen allerdings abführend wirken.

Xylit, auch Birkenzucker genannt, wird in der Regel nicht aus Birkenholz, sondern aus landwirtschaftlichen Reststoffen wie Maiskolbentrester, Stroh und aus Harthölzern gewonnen. Xylit ist nicht Karies fördernd und hat ca. die Hälfte der Kalorien des herkömmlichen Haushaltszuckers. Xylit belastet nicht den Insulinspiegel, kann in größeren Mengen allerdings abführend wirken.

Stevia oder “Stevia-Streusüße” bestehen hauptsächlich aus Erythrit oder dem Mehrfachzucker Maltodextrin. Sie enthalten kein “Stevia” in natürlicher Form. Im Vergleich zu Zucker hat Stevia etwa die 300-fache Süßkraft und einen lakritzartigen Eigengeschmack. Steviablätter stammen jedoch nicht aus der Region oder Europa. Der Transport der Rohstoffe belastet daher unnötig die Umwelt und das Klima. Es ist kein Einfluss auf den Blutzuckerspiegel bekannt und Stevia wirkt nicht karieserzeugend. Stevia ist fast kalorienfrei. [19]

### Appendix A.2. English Translation of the Information of the Four Sweeteners

Allulose is a natural calorie-free sugar. It is obtained by refining conventional beet sugar from regionally grown sugar beets. This is done with the help of enzymes. Nevertheless, allulose is a purely plant-based product. It has been proven to be tooth-friendly and is also suitable for diabetics, as it does not affect blood sugar levels or insulin levels. Unlike many other sugar alternatives, it has a taste typical of sugar and is well-tolerated.

Erythritol occurs naturally only in small quantities in fruit, vegetables, and cheese and is therefore mostly derived from industrial production. In various biotechnological processing steps involving fungi, erythritol is produced from carbohydrates such as glucose or sucrose. The sugar alternative does not promote caries and is calorie-free. Erythritol does not affect insulin levels, but in larger quantities it can have a laxative effect.

Xylitol, also known as birch sugar, is not usually made from birch wood but from agricultural residues such as corncob pomace, straw, and hardwoods. Xylitol does not promote tooth decay and has about half the number of calories of conventional household sugar. Xylitol does not affect insulin levels, but in larger quantities it can have a laxative effect.

Stevia or ‘stevia spread sweeteners’ consist mainly of erythritol or the polysaccharide maltodextrin. They do not contain ‘stevia’ in its natural form. Compared to sugar, stevia has about 300 times the sweetening power and a liquorice-like taste. However, stevia leaves are not grown regionally or in Europe. The transport of the raw materials therefore unnecessarily pollutes the environment and the climate. There is no known effect on blood sugar levels, and stevia does not cause caries. Stevia has almost no calories. [19]

## Appendix B

**Table A1:** Importance of characteristics in one’s own diet.

Characteristics	Mean	SD	Participants (%) Who Paid Much Attention to the Characteristics
Naturalness	3.96	1.08	36.2
Regional production	3.86	1.08	30.3
Low in sugar	3.84	1.12	34.5
Few additives	3.81	1.18	34.0
Few sweeteners	3.62	1.29	31.8
High in fiber	3.48	1.13	17.4
Low-calorie	3.19	1.20	13.2
High-protein (lots of protein)	2.68	1.31	9.2
Low-carb (few carbohydrates)	2.67	1.21	7.2
Keto (low carbohydrate, high fat)	2.27	1.28	8.4
Vegan	2.00	1.24	6.2

Note: SD = standard deviation. Question: ‘Do you pay attention to the following things in your diet?’ Scale from 1 = ‘I don’t pay attention at all’ to 5 = ‘I pay a lot of attention’.

## References

[1] World Health Organization (2016). Global Report on Diabetes.

[2] Branca F., Lartey A., Oenema S., Aguayo V., Stordalen G.A., Richardson R., Arvelo M., Afshin A. (2019). Transforming the food system to fight non-communicable diseases. BMJ.

[3] Ernst J.B., Arens-Azevêdo U., Bosy-Westphal A., de Zwaan M., Egert S., Fritsche A., Gerlach S., Hauner H., Heseker H., Koletzko B. (2019). Quantitative recommendation on sugar intake in Germany: Short version of the consensus paper by the German Obesity Society (DAG), German Diabetes Society (DDG) and German Nutrition Society (DGE). Ernährungs. Umsch..

[4] World Health Organization (2015). Guideline: Sugars Intake for Adults and Children.

[5] Kleis L.D., Schulte E.A., Buyken A.E. (2020). Reformulation across Europe: An overview on planned and implemented strategies in European countries other than Germany—Part 1. Ernährungs. Umsch. Int..

[6] Obesity Health Alliance (2021). Turning the Tide: A 10-Year Healthy Weight Strategy. http://obesityhealthalliance.org.uk/wp-content/uploads/2021/09/Turning-the-Tide-A-10-year-Healthy-Weight-Strategy.pdf.

[7] Bagus T., Roser S.A., Watzl B. (2016). Reformulierung von Verarbeiteten Lebensmitteln—Bewertungen und Empfehlungen zur Reduktion des Zuckergehalts: [Reformulation of Processed Foods—Assessments and Recommendations for Sugar Content Reduction], Karlsruhe. https://www.openagrar.de/receive/openagrar_mods_00043809.

[8] Mennella J.A., Beauchamp G.K. (1998). Early flavor experiences: Research update. Nutr. Rev..

[9] Liem D.G., de Graaf C. (2004). Sweet and sour preferences in young children and adults: Role of repeated exposure. Physiol. Behav..

[10] Shepherd R. (2002). Resistance to changes in diet. Proc. Nutr. Soc..

[11] Mortensen A. (2006). Sweeteners permitted in the European Union: Safety aspects. Food Nutr. Res..

[12] de Cock P., O’Donnell K., Kearsley M.W., Kearsley M.W. (2012). Erythritol. Sweeteners and Sugar Alternatives in Food Technology.

[13] Carocho M., Morales P., Ferreira I.C.F.R. (2017). Sweeteners as food additives in the XXI century: A review of what is known, and what is to come. Food Chem. Toxicol..

[14] Worsfold P., Townshend A., Poole C.F., Miró M. (2005). Encyclopedia of Analytical Science.

[15] Saraiva A., Carrascosa C., Raheem D., Ramos F., Raposo A. (2020). Natural Sweeteners: The Relevance of Food Naturalness for Consumers, Food Security Aspects, Sustainability and Health Impacts. Int. J. Environ. Res. Public Health.

[16] Younes M., Aquilina G., Engel K.-H., Fowler P., Frutos Fernandez M.J., Fürst P., Gürtler R., Gundert-Remy U., Husøy T., Manco M. (2020). Safety of a proposed amendment of the specifications for steviol glycosides (E 960) as a food additive: To expand the list of steviol glycosides to all those identified in the leaves of *Stevia rebaudiana* Bertoni. EFS2.

[17] Goyal S.K., Samsher, Goyal R.K. (2010). Stevia (*Stevia rebaudiana*) a bio-sweetener: A review. Int. J. Food Sci. Nutr..

[18] Ruiz-Ojeda F.J., Plaza-Díaz J., Sáez-Lara M.J., Gil A. (2019). Effects of Sweeteners on the Gut Microbiota: A Review of Experimental Studies and Clinical Trials. Adv. Nutr..

[19] Rosenplenter K., Nöhle U., von Rymon Lipinski G.-W. (2007). Handbuch Süßungsmittel: Eigenschaften und Anwendung.

[20] Izumori K. (2006). Izumoring: A strategy for bioproduction of all hexoses. J. Biotechnol..

[21] Zhang W., Yu S., Zhang T., Jiang B., Mu W. (2016). Recent advances in d-allulose: Physiological functionalities, applications, and biological production. Trends Food Sci. Technol..

[22] Matsuo T., Suzuki H., Hashiguchi M., Izumori K. (2002). D-psicose is a rare sugar that provides no energy to growing rats. J. Nutr. Sci. Vitaminol. J. Nutr. Sci. Vitam..

[23] Hossain A., Yamaguchi F., Matsuo T., Tsukamoto I., Toyoda Y., Ogawa M., Nagata Y., Tokuda M. (2015). Rare sugar D-allulose: Potential role and therapeutic monitoring in maintaining obesity and type 2 diabetes mellitus. Pharmacol. Ther..

[24] Southey F. (2021). Allulose Approval in Europe to be Sought by New Ingredients Consortium. Foodnavigator. https://www.foodnavigator.com/Article/2021/12/07/Allulose-approval-in-Europe-to-be-sought-by-new-ingredients-consortium.

[25] (2019). Studien-Report: Zuckeralternativen im Süßwarensegment.

[26] Pielak M., Czarniecka-Skubina E., Trafiałek J., Głuchowski A. (2019). Contemporary trends and habits in the consumption of sugar and sweeteners—A questionnaire survey among Poles. Int. J. Environ. Res. Public Health.

[27] Mintel (2017). Baking Provides Bright Spot in Canada’s Sugar Market. https://www.mintel.com/press-centre/food-and-drink/baking-provides-bright-spot-in-canadas-sugar-market.

[28] Sipple L.R., Racette C.M., Schiano A.N., Drake M.A. (2022). Consumer perception of ice cream and frozen desserts in the “better-for-you” category. J. Dairy Sci..

[29] Goodman S., Vanderlee L., Jones A., White C., Hammond D. (2021). Perceived Healthiness of Sweeteners among Young Adults in Canada. Can. J. Diet. Pract. Res..

[30] Farhat G., Dewison F., Stevenson L. (2021). Knowledge and Perceptions of Non-Nutritive Sweeteners within the UK Adult Population. Nutrients.

[31] Sylvetsky A.C., Greenberg M., Zhao X., Rother K.I. (2014). What parents think about giving nonnutritive sweeteners to their children: A pilot study. Int. J. Pediatr..

[32] Hu M., Li M., Jiang B., Zhang T. (2021). Bioproduction of D-allulose: Properties, applications, purification, and future perspectives. Compr. Rev. Food Sci. Food Saf..

[33] McFadden D., Zarembka P. (1974). Conditional logit analysis of qualitative choice behavior. Frontiers in Econometrics.

[34] Elshiewy O., Guhl D., Boztug Y. (2017). Multinomial logit models in marketing—From fundamentals to state-of-the-art. MAR.

[35] Lockshin L., Jarvis W., d’Hauteville F., Perrouty J.-P. (2006). Using simulations from discrete choice experiments to measure consumer sensitivity to brand, region, price, and awards in wine choice. Food Qual. Prefer..

[36] Jürkenbeck K., Spiller A. (2020). Importance of sensory quality signals in consumers’ food choice. Food Qual. Prefer..

[37] Rusmevichientong P., Jaynes J., Chandler L. (2021). Understanding influencing attributes of adolescent snack choices: Evidence from a discrete choice experiment. Food Qual. Prefer..

[38] Schulze M., Spiller A., Risius A. (2021). Do consumers prefer pasture-raised dual-purpose cattle when considering meat products? A hypothetical discrete choice experiment for the case of minced beef. Meat Sci..

[39] van Loo E.J., Caputo V., Nayga R.M., Meullenet J.-F., Ricke S.C. (2011). Consumers’ willingness to pay for organic chicken breast: Evidence from choice experiment. Food Qual. Prefer..

[40] van Loo E.J., Caputo V., Lusk J.L. (2020). Consumer preferences for farm-raised meat, lab-grown meat, and plant-based meat alternatives: Does information or brand matter?. Food Policy.

[41] Pabst E., Corsi A.M., Vecchio R., Annunziata A., Loose S.M. (2021). Consumers’ reactions to nutrition and ingredient labelling for wine—A cross-country discrete choice experiment. Appetite.

[42] Hein K.A., Jaeger S.R., Tom Carr B., Delahunty C.M. (2008). Comparison of five common acceptance and preference methods. Food Qual. Prefer..

[43] Meyerding S.G. (2016). Consumer preferences for food labels on tomatoes in Germany—A comparison of a quasi-experiment and two stated preference approaches. Appetite.

[44] Kozak M., Cliff M.A. (2013). Systematic comparison of hedonic ranking and rating methods demonstrates few practical differences. J. Food Sci..

[45] Lagerkvist C.J. (2013). Consumer preferences for food labelling attributes: Comparing direct ranking and best–worst scaling for measurement of attribute importance, preference intensity and attribute dominance. Food Qual. Prefer..

[46] Hebden L., Chan H.N., Louie J.C., Rangan A., Allman-Farinelli M. (2015). You are what you choose to eat: Factors influencing young adults’ food selection behaviour. J. Hum. Nutr. Diet..

[47] Lusk J.L., Briggeman B.C. (2009). Food values. Am. J. Agric. Econ..

[48] Román S., Sánchez-Siles L.M., Siegrist M. (2017). The importance of food naturalness for consumers: Results of a systematic review. Trends Food Sci. Technol..

[49] Drewnowski A., Mennella J.A., Johnson S.L., Bellisle F. (2012). Sweetness and food preference. J. Nutr..

[50] Deutsche Diabetes Gesellschaft (DDG) und Deutsche Diabetes-Hilfe (2021). Deutscher Gesundheitsbericht Diabethes 2021.

[51] Iida T., Kishimoto Y., Yoshikawa Y., Hayashi N., Okuma K., Tohi M., Yagi K., Matsuo T., Izumori K. (2008). Acute D-psicose admin-istration decreases the glycemic responses to an oral maltodextrin tolerance test in normal adults. Journal of Nutritional Science and Vitaminology. J. Nutr. Sci. Vitaminol..

[52] Noronha J.C., Braunstein C.R., Glenn A.J., Khan T.A., Viguiliouk E., Noseworthy R., Blanco Mejia S., Kendall C.W.C., Wolever T.M.S., Leiter L.A. (2018). The effect of small doses of fructose and allulose on postprandial glucose metabolism in type 2 diabetes: A double-blind, randomized, controlled, acute feeding, equivalence trial. Diabetes Obes. Metab..

[53] Stampa E., Schipmann-Schwarze C., Hamm U. (2020). Consumer perceptions, preferences, and behavior regarding pasture-raised livestock products: A review. Food Qual. Prefer..

[54] Burt B.A., Pai S. (2001). Sugar consumption and caries risk: A systematic review. J. Dent. Educ..

[55] Morozova Y., Misova E., Foltasova L., Sedlata-Juraskova E., Tyrda V. (2016). Food Components in Oral Health. Int. J. Pharm. Sci. Invent..

[56] Lim S.-L., Penrod M.T., Ha O.-R., Bruce J.M., Bruce A.S. (2018). Calorie Labeling Promotes Dietary Self-Control by Shifting the Temporal Dynamics of Health- and Taste-Attribute Integration in Overweight Individuals. Psychol. Sci..

